# Dose‐rate dependence and IMRT QA suitability of EBT3 radiochromic films for pulse reduced dose‐rate radiotherapy (PRDR) dosimetry

**DOI:** 10.1002/acm2.14229

**Published:** 2023-11-30

**Authors:** Ahtesham Ullah Khan, Jeff Radtke, Clifford Hammer, Julia Malyshev, Brett Morris, Carri Glide‐Hurst, Larry DeWerd, Wesley Culberson, Adam Bayliss

**Affiliations:** ^1^ Department of Medical Physics, School of Medicine and Public Health University of Wisconsin‐Madison Madison Wisconsin USA; ^2^ Department of Human Oncology, School of Medicine and Public Health University of Wisconsin‐Madison Madison Wisconsin USA

**Keywords:** dose‐rate dependence, EBT3 film, film dosimetry, IMRT QA, PRDR

## Abstract

**Background:**

Pulsed reduced dose rate (PRDR) is an emerging radiotherapy technique for recurrent diseases. It is pertinent that the linac beam characteristics are evaluated for PRDR dose rates and a suitable dosimeter is employed for IMRT QA.

**Purpose:**

This study sought to investigate the pulse characteristics of a 6 MV photon beam during PRDR irradiations on a commercial linac. The feasibility of using EBT3 radiochromic film for use in IMRT QA was also investigated by comparing its response to a commercial diode array phantom.

**Methods:**

A plastic scintillator detector was employed to measure the photon pulse characteristics across nominal repetition rates (NRRs) in the 5–600 MU/min range. Film was irradiated with dose rates in the 0.033–4 Gy/min range to study the dose rate dependence. Five clinical PRDR treatment plans were selected for IMRT QA with the Delta4 phantom and EBT3 film sheets. The planned and measured dose were compared using gamma analysis with a criterion of 3%/3 mm. EBT3 film QA was performed using a cumulative technique and a weighting factor technique.

**Results:**

Negligible differences were observed in the pulse width and height data between the investigated NRRs. The pulse width was measured to be 3.15 ± 0.01 μs and the PRF was calculated to be 3–357 Hz for the 5–600 MU/min NRRs. The EBT3 film was found to be dose rate independent within 3%. The gamma pass rates (GPRs) were above 99% and 90% for the Delta4 phantom and the EBT3 film using the cumulative QA method, respectively. GPRs as low as 80% were noted for the weighting factor EBT3 QA method.

**Conclusions:**

Altering the NRRs changes the mean dose rate while the instantaneous dose rate remains constant. The EBT3 film was found to be suitable for PRDR dosimetry and IMRT QA with minimal dose rate dependence.

## 1 INTRODUCTION

Pulse reduced dose‐rate radiotherapy (PRDR) is a promising treatment technique for re‐irradiation of recurrent disease.^1^, ^2^, ^3^, ^4^, ^5^, ^6^, ^7^ This technique involves delivering radiation at dose rates below 0.0667 Gy/min.^3^, ^4^, ^8^ Slowing the rate of daily radiation delivery is hypothesized to increase intrafraction sublethal damage repair and thus decrease late toxicity associated with re‐irradiation.^9^ It may also increase the therapeutic benefit by leveraging the hyper‐sensitivity of tumor cells at low dose rates of radiation.^8^, ^10^ Retrospective studies have demonstrated the safety and efficacy of this technique in multiple disease sites, showing encouraging overall survival outcomes in heavily pre‐treated pediatric and adult patients.^2^, ^7^, ^11^, ^12^

When PRDR was first proposed, conventional linear accelerators (linacs) were capable of delivering photon beams with a dose rate of 100−600 MU/min, which typically translated to dose rates of 1−6 Gy/min at the isocenter.^8^ For the PRDR technique, dose rates below 0.0667 Gy/min are desired since cell survival is significantly impacted at low dose rates.^13^ Therefore, the prescription fractional dose of 2 Gy is decomposed into at least 10 subfractions with doses < 0.2 Gy delivered in 3‐min intervals while the patient is on the table. This method ensures that a low dose is delivered to the tumor for each subfraction while retaining the same total fractional dose, and most importantly, the normal cells are exposed to substantially lower dose rates. More modern linacs, such as a Varian TrueBeam, can deliver a 6 MV filter flattened (FF) photon beam with dose rates well below 100 MU/min with the lowest being 5 MU/min. At the authors’ institution, a 6 MV photon beam is used for PRDR volumetric arc therapy (VMAT) delivery with dose rates ranging from 5−100 MU/min. Since this is a different delivery paradigm than was used in the prior studies, beam characteristics are investigated for these low dose‐rate irradiations to establish a PRDR program. Additionally, a suitable dosimeter is sought to study the dosimetric properties of the photon beam at low dose rates and to perform intensity‐modulated radiation therapy (IMRT) quality assurance (QA).

Due to the pulsed beam structure of medical linacs, dose rate can be classified as instantaneous dose rate, ${\dot{D}}_{p}$, or time‐averaged dose rate/mean dose rate, ${\dot{D}}_{m}$. The instantaneous dose rate is given as a ratio of dose‐per‐pulse to the pulse length, whereas the mean dose rate accounts for the time between the individual pulses. Thus, the instantaneous dose rate is always greater than the mean dose rate for pulsed beams. Radiation dosimeters may exhibit dependence on instantaneous dose rates and independence on mean dose rates or vice versa. It is pertinent to probe the pulse structure of the photon beam for the PRDR dose rates to better understand the relationship between the instantaneous dose rate and the mean dose rate. Plastic scintillator detectors (PSD) are suitable for the measurement of pulse repetition frequency (PRF), pulse with, and pulse height due to their prompt light emission and short fluorescence decay time.^14^

Radiochromic films, such as EBT3 film or EBT4 film, are suitable candidates for PRDR dosimetry due to their energy independence, angular independence, high spatial resolution, and 2D readout.^15^, ^16^, ^17^ Several investigations have previously evaluated the dose rate dependence of radiochromic films for conventional dose rates above 100 MU/min.^15^, ^18^, ^19^, ^20^, ^21^ However, to the authors’ knowledge, the dose rate dependence of EBT3 film has yet to be evaluated for PRDR photon beams delivered by a linac. Performing measurement‐based IMRT QA can also be challenging for PRDR treatment plans using detectors that are typically characterized for conventional dose rates. Therefore, the feasibility of using EBT3 film for performing IMRT QA must also be studied. Ma et al. previously evaluated the EDR2 film for IMRT QA of PRDR plans.^22^ They performed QA for four clinical treatment plans and found gamma pass rates over 95% for a 3%/3 mm criteria. It is of note that their work focused on linacs capable of generating dose rates above 100 MU/min. Thus, there is a need to evaluate radiochromic films below the 100 MU/min dose rate.

This work seeks to investigate some of the clinical challenges with reliable generation of PRDR plans including the impact of nominal dose rate on pulse characteristics of a 6 MV photon beam and characterization EBT3 film for PRDR dosimetry. The dose‐rate dependence of EBT3 film was evaluated for dose rates in the 5−600 MU/min range. Using EBT3 film and a diode‐array phantom, IMRT QA was performed for five clinical treatment plans.

## 2 MATERIALS AND METHODS

### 2.1 Beam pulse characteristics

**FIGURE 1:**
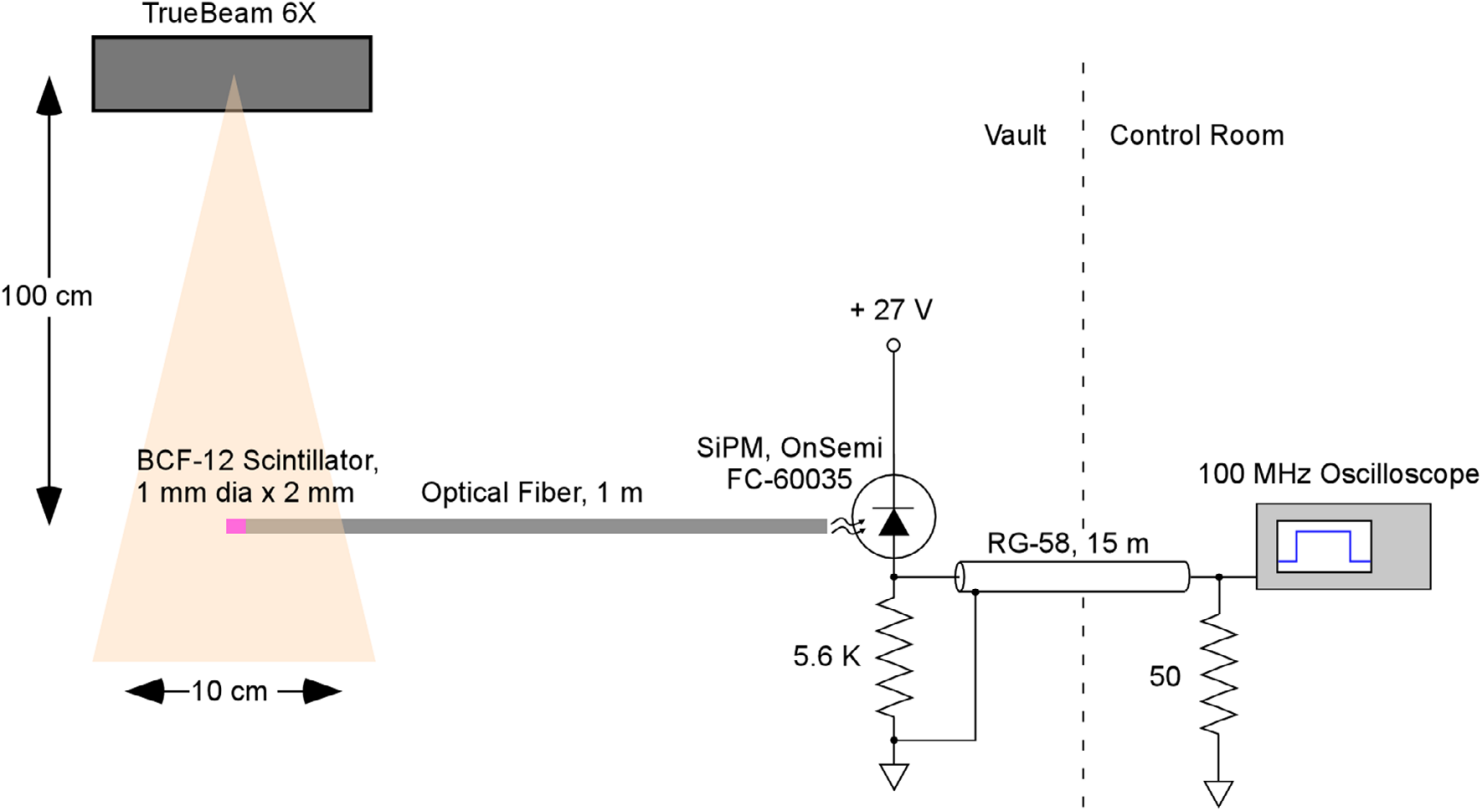
The irradiation setup for the scintillator connected to a silicon photomultiplier tube (SiPM) to measure the pulse characteristics.

All irradiations were performed using a 6 MV photon beam from a Varian TrueBeam (Palo Alto, CA) linac. Figure 1 shows the schematic of the setup used for scintillator irradiations. The gantry angle and collimator angle were set to 0^°^ with the field size set to 10 × 10 cm^2^. A plastic scintillator fiber (BCF‐12, Saint‐Gobain Crystals, Hiram OH) was placed on the treatment couch with the source‐to‐detector distance (SDD) of 100 cm with no buildup or backscatter material. This scintillator has an efficiency of ∼8000 photons/MeV with a peak emission of 435 nm and a decay time of 3.2 ns. The scintillator was coupled to a silicon photomultiplier tube (SiPM, MicroFC‐60035, Onsemi, Phoenix AZ), which was connected to an oscilloscope (Keysight DSOX1204A) placed outside the treatment vault. The field size and the geometrical setup was kept identical between these measurements to ensure a similar contribution from the Cerenkov signal to the total signal across all dose rates. The SiPM employed in this work consisted of 18980 microcells, each with an avalanche photodiode and series resistor. These microcells are connected in parallel and represented in Figure 1 as a composite, reverse‐biased diode. A single photon can trigger a charge multiplication avalanche in a microcell photodiode. The avalanche pulse is limited (quenched) by the voltage drop across the microcell resistor, which is proportional to avalanche current. The pulse observed with the oscilloscope is the sum of these avalanches. Since the avalanche gain is about $3\;\times \;10^{6}$, amplification is not required to observe pulses across the 50‐ohm cable termination resistance with an oscilloscope. Avalanche rise time was governed by the scintillation light decay time, since the intrinsic SiPM rise time was 1 ns. The microcell avalanche/quench cycle occurs in less than 100 ns. The optical fiber is positioned to illuminate the entire face of the SiPM, so the large number of microcells yields a dynamic range which exceeds three orders of magnitude. An air gap between the optical fiber and SiPM also serves to attenuate the scintillation light and avoid triggering most of the microcells during the 100 ns quench cycle, thus precluding SiPM saturation. The pulses observed by the oscilloscope thus reflect the temporal profile of the linac output radiation. The utilized SiPM includes a “fast” output, which is capacitively coupled to all the microcells. Although this output channel is good for timing purposes, it provides a derivative of the illumination pulse, and lacks information about light intensity during the pulse. The fast output was not used for measurements in this study.

For each nominal dose rate in the 5−600 MU/min range, 1000 MUs were delivered, and the pulse height, pulse width, and pulse separation were measured. Averaging acquisition mode was utilized on the oscilloscope and an average of 512 pulses was captured and exported to MATLAB v2020b (Natick, MA) for analysis. The full width at half maximum (FWHM) was quantified as the pulse width and the mean of the uniform region was defined to be the pulse height. The uniform region was defined to be the region between the peak after the rise of the pulse and the peak before the fall of the pulse. Two vertical cursors on the oscilloscope were used to measure the pulse separation. The PRF was calculated by taking the inverse of the pulse separation.

### 2.2 Dose rate dependence of EBT3 film

Gafchromic EBT3 films (Ashland, Bridgewater, NJ) were irradiated using a 6 MV photon beam from a Varian TrueBeam linac. Both gantry and collimator angles were set to 0^°^ throughout these irradiations. A 10 × 10 cm^2^ field size was selected with a source‐to‐surface distance (SSD) of 100 cm. A 10 cm depth and 10 cm backscatter were provided using 30 × 30 cm^2^ solid water slabs (Gammex Inc, Middleton, WI). The 20 × 25 cm^2^ film sheets (lot #05112103) were cut into 5 × 5 cm^2^ squares using a laser cutter (Boss laser, Sanford, FL) and placed between the solid water slabs. Throughout this work, nitrile gloves were worn when handling the film. All film sheets were scanned prior to irradiations using a flatbed scanner (Epson 10000XL, Los Alamitos, CA). A 48‐bit (16‐bit per color) pixel depth was used with a 72 dpi (0.35 × 0.35 mm^2^ pixel size). Film sheets were scanned using the same settings at least 24 h following the irradiations. An opaque mask was used to surround the film sheets during the scanning process to reduce the lateral light scattering effects. The net optical density, $OD_{\mathrm{net}}$, was calculated by

(1) $$O\;D_{\mathrm{net}}=\log_{10}\left(\frac{I_{\mathrm{unexposed}}-I_{\mathrm{bkg}}}{I_{\mathrm{exposed}}-I_{\mathrm{bkg}}}\right)\;$$

(2) $$\begin{array}{ccc} & & \sigma_{OD_{\mathrm{net}}}=\frac{1}{ln10}\\ & & \;\sqrt{\frac{{\left(\sigma_{I_{\mathrm{unexposed}}}\right)}^{2}+{\left(\sigma_{I_{\mathrm{bkg}}}\right)}^{2}}{{\left(I_{\mathrm{unexposed}}-I_{\mathrm{bkg}}\right)}^{2}}+\frac{{\left(\sigma_{I_{\mathrm{exposed}}}\right)}^{2}+{\left(\sigma_{I_{\mathrm{bkg}}}\right)}^{2}}{{\left(I_{\mathrm{exposed}}-I_{\mathrm{bkg}}\right)}^{2}}} \end{array}$$

where *I*_unexposed_ is the mean pixel value of the pre‐scanned film, *I*_exposed_ is the mean pixel value of the irradiated film, *I*_bkg_ is the mean pixel value of the background film, and σ is the associated uncertainty.^23^ Only the red channel was considered in this work since it has the highest sensitivity to absorbed dose.^20^ Pixel values within 1 cm from the edges of the film were not included in the mean values to avoid effects from the cut at the edge.

Two sets of irradiations were performed with a total delivered absorbed dose of approximately 0.2 and 1 Gy, respectively, with dose rates in the 5−600 MU/min range, translating to 0.033–4 Gy/min dose rates. The MU calculation was performed using the RadCalc software (LAP GmbH Laser Applications, Austin, TX). A correction factor was applied to account for the material differences between water and solid water. The scanned images were imported in MATLAB 2020b (Natick, MA) and the net optical density was calculated following equation 1.

### 2.3 Planning methodology

Implementation of a PRDR program for VMAT can be challenged by machine deliverability. Ideally, the meter rate for a PRDR plan would be adjusted to ensure 6.67 cGy/min to the mean target dose per Ma et al.^5^ In Elekta systems like the Infinity series, equipped with the Integrity control system, meter rates are continuously variable enabling the meter rate to be programmed to achieve a dose rate adjusted beam.^24^, ^25^ Varian TrueBeam systems use binned dose rates varying between 5 and 600 MU/min on a flattened field. While the lowest possible meter setting (5 MU/min) would be sufficient to ensure a delivery at less than nominal PRDR dose rate, long arc delivery times create slow multileaf collimator (MLC) motion causing positioning drive motors to fail. These failures necessitated frequent reinitialization of the MLC positions or replacement of multiple motors at once. The authors have found that the minimum meter rate to avoid MLC errors is 10 MU/min which often exceeds the nominal PRDR dose rate for a given arc. Our current clinical practice ensures upper bounds on individual beams by dividing with them into PRDR subfractions that are delivered every 3 min at 100 MU/min. The beams are modulated using static gantry step and shoot (SNS) IMRT. Post‐optimization, each beam is divided into subfractions such that target doses of 0.2 Gy mean and 0.4 Gy maximum (0.03 cc) are not exceeded, then monitor units are divided evenly amongst subfractions. Modulation is reduced through limits on MU per control point (6 MU/min) and minimum control point area (< 4 cm^2^ per control point) to make them more robustly divisible into subfractions. Using an upper bound instead of the nominal PRDR dose rate leads to increased treatment times in a treatment that can easily exceed 45 min.

To address this issue, we have developed an alternative approach that enables subfraction‐based VMAT delivery at higher meter rates. Our approach uses Python code developed in‐house coupled with optimization features available in RayStation 10 to generate high‐quality VMAT plans for each site commonly treated in our clinic with PRDR, with almost no user intervention. By using this approach, we can deliver radiation therapy more efficiently while maintaining high‐quality treatment plans. The approach still relies on subfractions of 0.2 Gy delivered every 3 min until improvements in MLC motor design enable a lower leaf speeds. All plans tested in this study employ this method.

### 2.4 IMRT QA

The calibration curve was made using the setup described in section 2.2. The net OD was calculated for 5 × 5 cm^2^ film squares irradiated with absorbed dose of 0.03, 0.05, 0.10, 0.15, 0.20, 0.25, 0.30, 0.50, 0.75, 1.00, 1.50, 2.00, and 3.00 Gy. The dose rate was set to 100 MU/min for these irradiations. The sensitometry curve for the EBT3 film was fitted using a third‐order polynomial and a relationship was established to calculate the absorbed dose from the measured net OD. It is worth noting that the constructed calibration curve was lot‐specific to account for the variation in response between different film lots.

Several approaches can be taken to perform IMRT QA for PRDR treatment plans. Measurements can be performed on a composite basis by delivering all arcs. However, this method leads to measuring dose from the same beam set multiple times due to copied beams during the PRDR planning process. This method can be redundant and reduce the efficiency of the QA process. Alternatively, unique beams can be delivered, and the composite dose can be reconstructed using weighting factors based on the number of times a given beam was copied. This method reduces the total number of beams that must be delivered; however, it increases the number of film sheets required for QA. Each film sheet must be positioned inside the treatment vault for a given unique beam leading to an increased setup time. Additionally, the individual beams deliver an absorbed dose of < 0.2 Gy, which leads to a lower signal‐to‐noise ratio (SNR) and higher uncertainty in the measured dose relative to the composite dose method.

Five IMRT treatment plans as listed in Table 1 were selected for QA using EBT3 film and Delta4 diode‐array phantom. All plans were created using in‐house automated VMAT PRDR planning scripts that generate planning structures, load beams, define clinical goals, and import elements of the objective function. The scripting then loads and optimizes arcs through site‐specific workflows. For brain PRDR planning, a coplanar field and vertex field are loaded into separate “beam sets” which have independent inverse planning objectives and combined plan objectives. For thoracic irradiations, only coplanar arcs are used. Arcs are co‐optimized with each beam contributing mean beam dose of 0.2 Gy/subfraction where the total number of subfractions, $N_{sfx}$, is:

(3) $$N_{sfx}=N_{fx}\;\times \frac{D_{fx}}{D_{sfx}}\times \frac{1}{N_{arcs}}$$

where $N_{fx}$ is the number of fractions, $D_{fx}$ is the dose per fraction, $D_{sfx}$ is the dose per subfraction, and $N_{arcs}$ is the number of arcs. When optimization is complete, the two fully optimized arcs are combined and used as contributing half the planned dose for half the total number of subfractions. These arcs are fixed, and the remaining arcs are optimized using the reversed coplanar and vertex arcs. Thus, four arcs are optimized and copied until the overall number of subfractions per fraction is met. Any remaining subfractions from odd $N_{sfx}$ are independently optimized. Clinically, a subfraction (or VMAT arc) is then delivered every 3 min. By individually optimizing each arc to delivers a mean dose to the target of exactly 0.2 Gy the result is superior to the SNS IMRT method by lowering overall treatment times on average by 12 min (4 subfractions), and eliminating modulation restrictions.

**TABLE 1:** Treatment site, number of beams, beam ID with the associated weighting factor, and maximum dose for each unique beam. The weighting factor reflects the number of repetitions for each beam in a given fraction.

Case index	Treatment site	Beam ID (weighting factor)	Maximum dose (Gy)
1	Glioblastoma (10 beams)	#1 (×3), #2 (×2), #6 (×3), #7 (×2)	0.17, 0.18, 0.18, 0.18
2	Whole brain (14 beams)	#1 (×4), #2 (×3), #8 (×4), #9 (×3)	0.15, 0.16, 0.15, 0.15
3	Whole brain with hippocampal avoidance (15 beams)	#1 (×4), #2 (×4), #9 (×3), #10 (×3), #15 (×1)	0.25, 0.33, 0.26, 0.25, 0.26
4	Esophagus (10 beams)	#1 (×5) and #2 (×5)	0.19 and 0.19
5	Breast (10 beams)	#1 (×4) and #2 (×6)	0.18 and 0.18

Table 1 lists the different properties of the five PRDR treatment plans chosen for this study. The total number of beams ranged from 10−15 with each plan containing 2−5 unique beams. The maximum dose per beam varied between 0.15 and 0.26 Gy. The TomoTherapy “cheese” phantom, that is included with the TomoTherapy (Accuray, Sunnyvale CA) machine, and the Delta4 Phantom+ were employed for IMRT QA. The Delta4 phantom is a cylindrical (22 cm diameter and 40 cm thickness) PMMA phantom with 1069 cylindrical (2 mm diameter and 0.05 mm thickness) p‐type silicon diodes embedded inside it. The diodes are centered on the coronal and the sagittal planes with a 5 mm detector spacing in the central 6 × 6 cm^2^ area. Outside the high‐resolution central area, the detector spacing is 10 mm. The TomoTherapy phantom is constructed into a cylinder (30 cm diameter and 18 cm thickness) using solid water. The phantom has drilled holes on each circular surface of the cylinder to accommodate ion chambers and cylindrical materials with known electron densities. Film sheets can be placed between the two semi‐cylindrical halves of the phantom. Dose in the coronal or sagittal planes can be sampled by rotating the phantom around its long axis. Two QA plans, one for Delta4 and one for the TomoTherapy phantom, were created in RayStation v11 (RaySearch, Stockholm, Sweden) for each treatment plan. The absorbed dose was calculated on the CT images of the two phantoms using a grid size of 2 × 2 × 2 mm^3^. The QA plans were exported, and the resulting DICOM plan files were imported into MATLAB for analysis.

Irradiations were performed using a TrueBeam STx linac with the Delta4 phantom and the TomoTherapy phantom. Absorbed dose in the coronal plane was sampled using 20 × 25 cm^2^ EBT3 film sheets. Prior to irradiations, the irradiated and background film sheets were scanned, and the images were stored. A background film sheet was scanned as well. At least 24 h post‐irradiation, the film sheets were scanned, and the resulting images were imported into MATLAB. A 3 × 3 median filter was applied to the images to reduce random noise. The net OD was calculated for each pixel using the pre‐irradiation and post‐irradiation scanned images. Conversion from net OD to absorbed dose was performed using the polynomial fit coefficients from the calibration curve. The pixel size was rescaled to match the TPS pixel size of 2 × 2 mm^2^. Rigid registration was performed between the film dose and the TPS dose to align the two 2D dose distributions. The measured absorbed dose was compared with the TPS dose using gamma analysis with a criterion of 3%/3 mm and a 10% threshold.^26^

The EBT3 film results for the composite QA method and the weighting factor method were also compared. For a given treatment plan, the cumulative IMRT PRDR QA technique involved irradiating a single film sheet with all the beams. Contrarily, the weighting factor‐based IMRT PRDR QA technique required grouping the beams with the same weight together and irradiating them using a single sheet. Therefore, the number of irradiated film sheets depends on the number of distinct weights in each treatment plan. For example, case 3 required three film sheets with the first sheet irradiated with beam#1 and beam#2, the second sheet irradiated with beam#9 and beam#10, and the third sheet irradiated with beam#15. The weighting factors were applied following the measurements to reconstruct the cumulative absorbed dose.

## 3 RESULTS

### 3.1 Beam pulse characteristics

**FIGURE 2:**
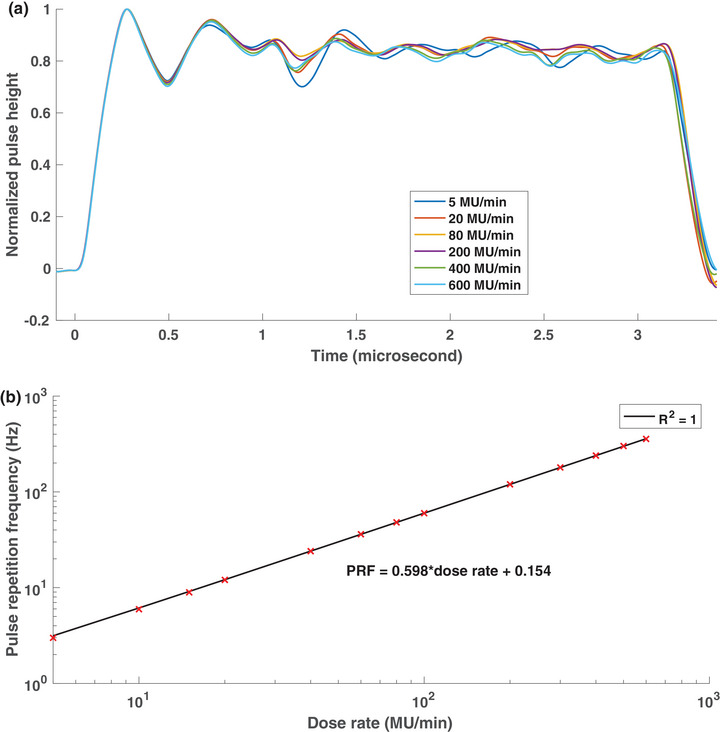
(a) Single pulse structure of a 6 MV photon beam from a Varian TrueBeam linac for dose rates in the 5−100 MU/min range. Each pulse was normalized by the maximum pulse height. (b) The relationship between the nominal dose rate and the pulse repetition frequency for the 6 MV photon beam.

The pulse shape, measured using a PSD, for dose rates ranging from 5 MU/min to 600 MU/min is shown in Figure 2a. Random noise was noted in the pulse height due to a low SNR for the single pulse data. Several ripples were noted in the pulse height, especially at the beginning of the pulse. The pulse width FWHM was measured to be 3.15 ± 0.01 μs for all investigated dose rates. A percent standard deviation of 0.42% was calculated in the pulse width measurements. The variation in the mean pulse height between different dose rates was found to be 0.53%. It may be concluded from these results that there is no difference in the pulse width or height between different dose rates. Figure 2b displays the PRF as a function of the nominal dose rate. The maximum PRF for the 6 MV photon beam was measured to be 357 Hz and the PRF for dose rates below 100 MU/min was determined to be < 60 Hz. A linear relationship was noted between the PRF and the nominal dose rate with an *R*2 of 1.00. Therefore, it can be concluded that the instantaneous dose rate remains constant while the mean dose rate varies when changing the nominal dose rate settings.

### 3.2 Dose rate dependence of EBT3 film

**FIGURE 3:**
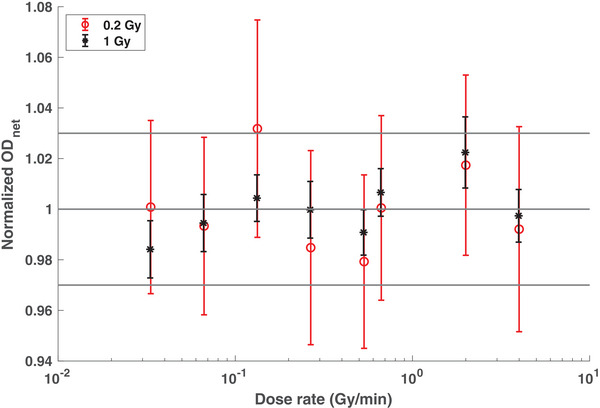
Dose rate dependence of EBT3 film in a 6 MV photon beam measured for delivered absorbed dose of 0.2 Gy and 1.0 Gy. The measured net OD was normalized to the mean net OD for a given absorbed dose. The error bars correspond to 1σ uncertainty. The horizontal lines correspond to a 3% agreement level with unity.

The net ODs for various dose rates, normalized by the mean net OD, are shown in Figure 3. No clear trend was noted between the measured response and the investigated dose rates. A variation in the normalized $OD_{net}$ across the investigated dose rate, in the form of percent standard deviation, was calculated to be 1.70% and 1.20% for the 0.2 Gy and the 1.0 Gy absorbed dose, respectively. It is noteworthy that the average 1σ uncertainty was 1.07% for film irradiated with 1 Gy dose, while the average uncertainty for the 0.2 Gy film was much higher at 3.71%. The random noise in the film pixels increases at low doses leading to higher uncertainties. The largest deviation from the mean net OD was calculated to be 3.20% and 2.20% for the 0.2 Gy and the 1.0 Gy dose, respectively. Thus, considering the uncertainty in the data, EBT3 film was found to be dose rate independent to within 3.0%.

### 3.3 IMRT QA

**FIGURE 4:**
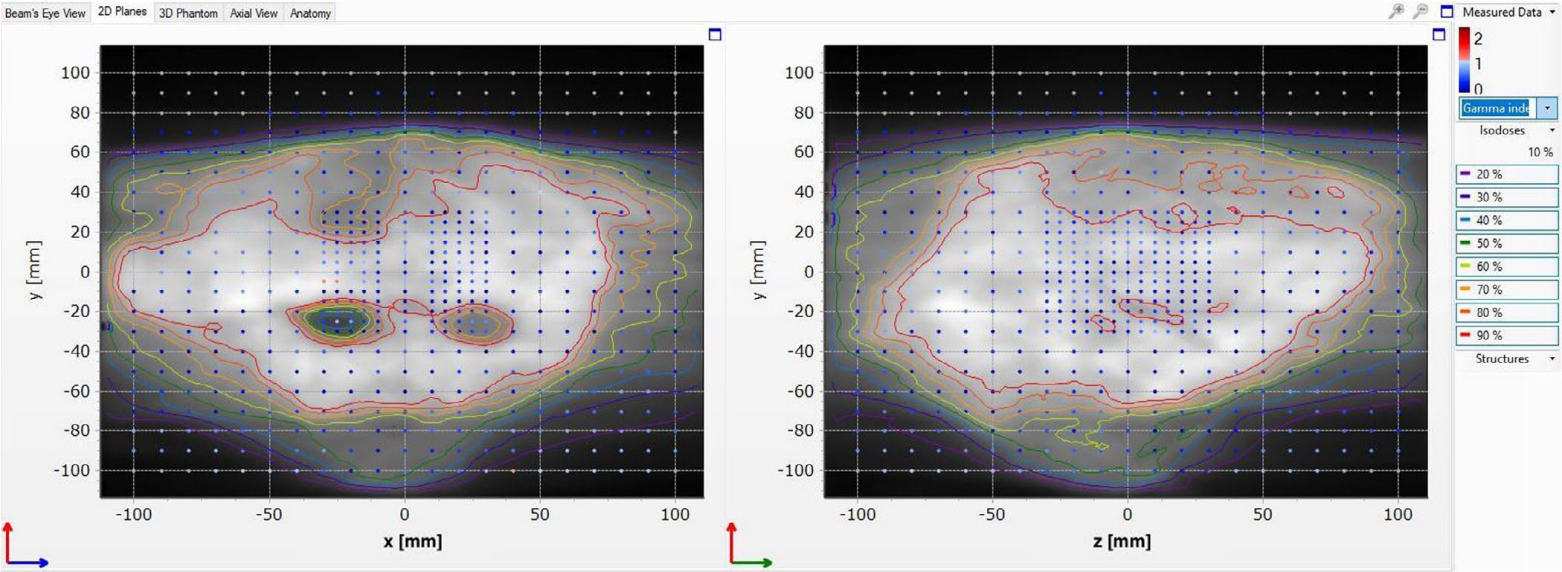
Isodose contours for case 3 planned 2D coronal (left) and sagittal (right) anatomical dose planes along with gamma indices for the measured dose using the Delta4 phantom. The gamma indices > 1 appear orange/red in color and indices < 1 appear blue.

The measured absorbed dose distributions for the coronal and sagittal planes were compared with the planned dose using the Delta4 diode array phantom. An example of the measured dose and the planned dose is shown in Figure 4 for case 3 (whole brain with hippocampal avoidance) along with the associated gamma indices. Excellent agreement was found between the cumulative planned and the measured dose distributions with the gamma pass rates (GPRs) of 100% and 99.5% for all cases using 3%/3 mm and 3%/2 mm criteria, respectively. The Delta4 phantom was deemed suitable for PRDR IMRT QA.

**FIGURE 5:**
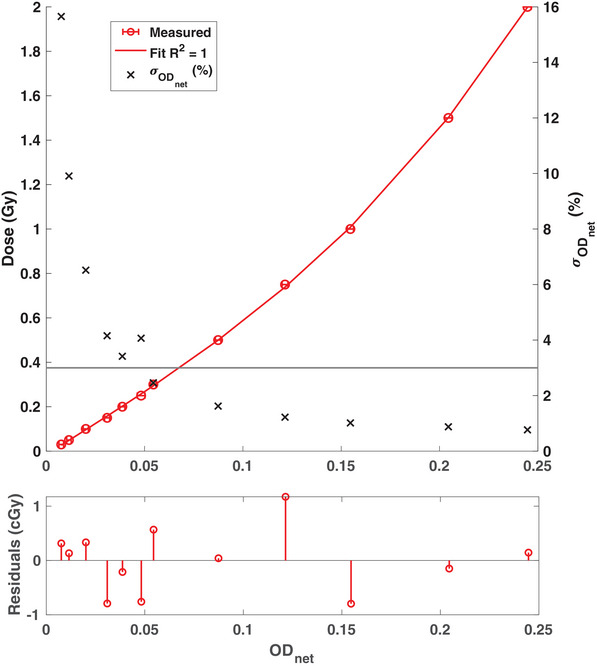
The sensitometry curve for the EBT3 film for absorbed dose in the 0.03–2.00 Gy range. The measured data fitted to a third‐order polynomial led to an *R* ^2^ of 1.00. The error bars on the measured net OD are smaller than the marker size. The uncertainty in the net OD was also plotted for the delivered dose $\sigma_{OD_{net}}\left(\%\right)$.

**FIGURE 6:**
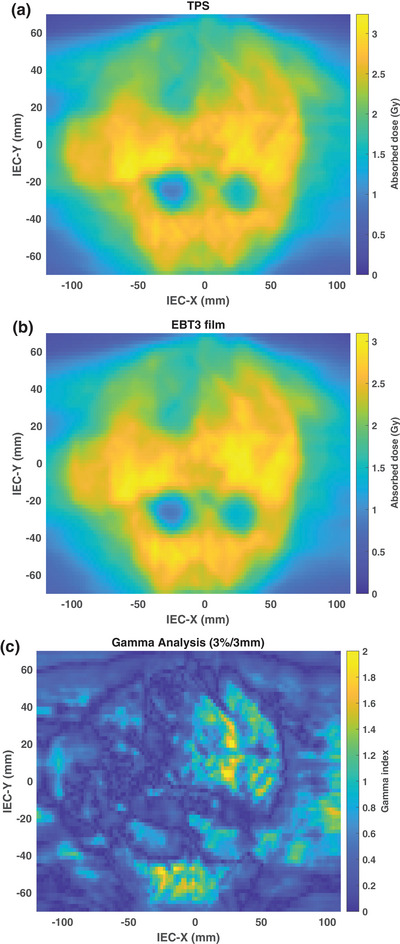
(a) the TPS planned dose and (b) the EBT3 measured cumulative dose for the coronal plane of whole brain with hippocampal avoidance case. (c) Gamma index using a 3%/3 mm criterion.

The calibration curve for the film lot used for IMRT QA is shown in Figure 5. The sensitivity of the EBT3 film was found to decrease with increasing dose. Using a third‐order polynomial fit, the residual error was noted to be within 1.1 cGy. For absorbed doses below 0.3 Gy, the net OD remained within 0.06 while the uncertainty increased drastically above 3%. The planned and measured 2D dose distributions using EBT3 film for case 3 are shown in Figure 6. Overall good agreement was found between the two dose distributions with a resulting GPR of 93.0% using a 3%/3 mm criteria. Table 2 compares the planned and measured dose distributions using EBT3 film by compiling the GPRs for the cumulative irradiation method and the weighting factor method. All cumulative GPRs were noted to be above 90% with the highest GPR of 98.1%. For cases 1−4, the weighting factor method resulted in GPRs above 90%. Additionally, the weighting factor GPRs were found to be larger than the cumulative GPRs for two out of five cases. However, a GPR of 80.4% was observed for case 5 when the QA was performed using the weighting factor technique. It is noteworthy that each film sheet was irradiated with a maximum dose of 0.18 Gy for this case when using the weighting factor method. A discussion of possible reasons behind the differences in the GPRs between the two methods is included in the following section.

**TABLE 2:** Gamma pass rates for the planned dose and the measured dose using EBT3 film sheets.

Case	Cumulative gamma pass rate (3%/3 mm)	Weighting factor gamma pass rate (3%/3 mm)
Glioblastoma	98.1	90.6
Whole brain	90.9	94.1
Whole brain with hippocampal avoidance	93.0	94.9
Esophagus	97.6	92.8
Breast	94.4	80.4

## 4 DISCUSSION

This work characterized the pulse structure of a clinical 6 MV photon beam using a PSD for dose rates typically used for PRDR treatments. The impact of changing the nominal repetition rate (NRR) on instantaneous and mean dose rates was investigated. The dose rate dependence of EBT3 film was studied for dose rates in the 0.033–4 Gy/min range. Placing EBT3 film in a TomoTherapy phantom and using a commercial diode array phantom, IMRT QA was performed for five clinical cases. The cumulative IMRT QA technique was compared with the weighting factor technique for EBT3 film irradiations.

Since the pulse height and the pulse width remained identical across the investigated NRR, the instantaneous dose rate was determined to be constant for the 6 MV photon beam. Therefore, the dose rate dependence of any detector must be studied in the context of the mean dose rate only. Small variations were noted in the pulse heights for different NRRs which are partially attributed to the random noise in the signal. The ripple effects observed in the single pulse structure of the beam reflect the RF pulse shape of the modulator, that is, klystron current. The PSD used in this work displayed excellent time resolution while the rise and decay time of the individual pulses was dictated by the charge clearance in the SiPM. The nominal decay time of the BCF‐12 PSD was quoted to be 3.2 ns by the manufacturer, which is sufficient to realize the individual pulses from the linac. However, the sub‐pulses were not resolved in this work due to their shorter separation time, which is on the order of picoseconds.

The EBT3 film displayed dose rate independence within 3% across the investigated mean dose rates. Karsch et al. found a dose rate dependence of up to 5% for the EBT film, a previous version of the EBT3 film, by varying the instantaneous dose rates from $2\;\times \;10^{8}$ Gy/s to $3\;\times \;10^{9}$ Gy/s.^19^ The work of Jaccard et al. previously reported dose rate independence of EBT3 film in electron beams for instantaneous dose rates in the $7\;\times \;10^{3}-8\;\times \;10^{6}$ Gy/s range and mean dose rates in the 0.55–4.4 Gy/min range.^18^ Similarly, Ataei et al. found dose rate independence of EBT3 film when irradiated with a 6 MV photon beam delivering dose rates of 2 Gy/min and 4 Gy/min (Ataei et al 2019). A study by Borca et al. also reported dose rate independence for EBTT3 film for dose rates in the 100−600 MU/min range.^20^ However, these studies only investigated dose rate dependence for mean dose rates above the PRDR threshold of 0.0667 Gy/min. This study extends their work by studying the film response for dose rates down to 0.033 Gy/min.

The usage of the Delta4 diode array phantom for PRDR IMRT QA was previously studied by Geurts.^28^ For both static and step‐and‐shoot IMRT plans, excellent agreement was found between the diodes and ion chambers. This study corroborated their results and deemed Delta4 a suitable detector for PRDR IMRT QA. The Delta4 phantom was found to exhibit negligible mean dose rate dependence with GPRs above 99% for all five clinical plans utilized in this study. Previously, Ma et al. studied the suitability of EDR2 film for IMRT QA of PRDR plans with NRRs above 100 MU/min.^22^ For the four treatment sites investigated, GPRs above 95% were reported for the film. The work of Borca et al. characterized EBT3 film for IMRT QA of head & neck and pelvis plans with NRR above 100 MU/min.^20^ Their work reported GPRs above 97.0% for a 3%/3 mm criteria. This study builds on these works by investigating the feasibility of employing EBT3 film for IMRT PRDR QA with NRRs below 100 MU/min. The GPRs reported in this work were noted to be lower than the rates reported by Borca et al.^20^ However, the cumulative QA method led to GPRs above 90% for all investigated treatment sites.

The weighting factor QA method using EBT3 films was found to be suitable only when the individual film sheets were irradiated with a minimum dose above 0.3 Gy. As demonstrated in Figure 5, the uncertainty increases rapidly as absorbed dose decreases below 0.3 Gy. Therefore, significant noise was observed in the dose measured by individual film sheets for case 5. A similar trend was noted in the dose measured by the film sheet irradiated with case 3 beam#15. However, the overall GPR remained above 94% for this case due to the low weighting factor of this beam. The weighting factor method outperformed the cumulative method for cases 2 and 3. It is hypothesized that utilizing a new film sheet and re‐aligning the film for each distinct weight allows the reduction of positional offsets in the measurement setup. Whereas, in the case of cumulative QA, any initial positional offset in the measurement geometry persists in all beams. However, the cumulative method might have better detectability for any beam‐dependent shifts. Since only five treatment plans were included for IMRT QA in this study, this can be considered a limitation of this work. Another limitation of this study includes utilizing a 3%/3 mm gamma criteria for the Delta4 phantom instead of a stricter criterion. In the future, as PRDR becomes more prevalent, a large dataset of IMRT QA results will be studied to corroborate the conclusions drawn in this work.

As clinical outcomes continue to improve for cancer patients, reirradiation is likely to become increasingly utilized in patient care. PRDR may provide a unique treatment technique to increase the safety window for high dose reirradiation. Here, we demonstrate a robust IMRT QA process to help widely implement this technique in radiotherapy clinics.

## 5 CONCLUSIONS

No differences were found in the pulse height or length for a pulsed 6 MV photon beam across the NRRs of 5−600 MU/min. Therefore, the instantaneous dose rate remained constant while the mean dose decreased when reducing the NRR below 100 MU/min for PRDR treatments. The EBT3 film was found to be dose rate independent within 3% for dose rates in the 0.033–4.0 Gy/min range. The Delta4 phantom exhibited much higher GPRs than the EBT3 film. The cumulative QA method for the EBT3 film resulted in GPRs above 90%. The weighting factor QA method reduced the total irradiation time. However, this method must be avoided for absorbed dose below 0.3 Gy due to significant statistical noise noted in the EBT3 film.

## AUTHOR CONTRIBUTIONS

Ahtesham Khan, Brett Morris, and Adam Bayliss contributed to the preparation of the manuscript. Jeff Radtke fabricated the PSD apparatus used to measure the PRF of the linear accelerator (linac). He also assisted with the data collection and analysis. Clifford Hammer and Julia Malyshev assisted with the film calibration, data collection, and film analysis. Carri Glide‐Hurst and Wesley Culberson provided the scientific direction. Larry DeWerd, Adam Bayliss, Wesley Culberson, and Carri Glide‐Hurst supervised the work. Ahtesham Khan performed the experiments and gathered data. All authors analyzed the data and participated in the discussion.

## CONFLICT OF INTEREST STATEMENT

Ahtesham Ullah Khan, Jeff Radtke, Clifford Hammer, Julia Malyshev, Brett Morris, Larry DeWerd, Wesley Culberson, and Larry DeWerd have nothing to disclose. Adam Bayliss reports research collaborations with GE Healthcare. Carri Glide‐Hurst reports research collaborations with GE Healthcare and Modus Medical. Research partially supported by the National Cancer Institute of the National Institutes of Health under Award Number R01HL153720. The content is solely the responsibility of the authors and does not necessarily represent the official views of the National Institutes of Health.

## Data Availability

The data that support the findings of this study are available from the corresponding author upon reasonable request.
